# Structural Abnormalities of the Optic Nerve and Retina in Huntington’s Disease Pre-Clinical and Clinical Settings

**DOI:** 10.3390/ijms23105450

**Published:** 2022-05-13

**Authors:** Iwona Mazur-Michałek, Katarzyna Kowalska, Daniel Zielonka, Marta Leśniczak-Staszak, Paulina Pietras, Witold Szaflarski, Mark Isalan, Michal Mielcarek

**Affiliations:** 1 Department of Histology and Embryology, Poznan University of Medical Sciences, 61-701 Poznan, Poland; imazur.michalek@gmail.com (I.M.-M.); kkowalsk@ump.edu.pl (K.K.); marta.m.lesniczak@gmail.com (M.L.-S.); paulinapietras29@gmail.com (P.P.); witold.szaflarski@gmail.com (W.S.); 2 Department of Public Health, Poznan University of Medical Sciences, 61-701 Poznan, Poland; daniel.zielonka@gmail.com; 3 Department of Life Sciences, Imperial College London, Exhibition Road, London SW7 2AZ, UK; m.isalan@imperial.ac.uk; 4 Imperial College Centre for Synthetic Biology, Imperial College London, London SW7 2AZ, UK

**Keywords:** Huntington’s disease, ocular neurodegeneration, optic nerve, optical coherence tomography, transmission electron microscopy, mouse models

## Abstract

Huntington’s disease (HD) is a fatal neurodegenerative disorder caused by a polyglutamine expansion in the huntingtin protein. HD-related pathological remodelling has been reported in HD mouse models and HD carriers. In this study, we studied structural abnormalities in the optic nerve by employing Spectral Domain Optical Coherence Tomography (SD-OCT) in pre-symptomatic HD carriers of Caucasian origin. Transmission Electron Microscopy (TEM) was used to investigate ultrastructural changes in the optic nerve of the well-established R6/2 mouse model at the symptomatic stage of the disease. We found that pre-symptomatic HD carriers displayed a significant reduction in the retinal nerve fibre layer (RNFL) thickness, including specific quadrants: superior, inferior and temporal, but not nasal. There were no other significant irregularities in the GCC layer, at the macula level and in the optic disc morphology. The ultrastructural analysis of the optic nerve in R6/2 mice revealed a significant thinning of the myelin sheaths, with a lamellar separation of the myelin, and a presence of myelonoid bodies. We also found a significant reduction in the thickness of myelin sheaths in peripheral nerves within the choroids area. Those ultrastructural abnormalities were also observed in HD photoreceptor cells that contained severely damaged membrane disks, with evident vacuolisation and swelling. Moreover, the outer segment of retinal layers showed a progressive disintegration. Our study explored structural changes of the optic nerve in pre- and clinical settings and opens new avenues for the potential development of biomarkers that would be of great interest in HD gene therapies.

## 1. Introduction

Huntington’s disease is a fatal, autosomal dominant condition affecting movement and cognitive abilities [1]. This neurodegenerative disease is caused by a CAG DNA-repeat expansion (typically over ~40 CAGs) in the huntingtin gene (HTT). The CAGs are translated into a polyglutamine stretch within the huntingtin protein (HTT), leading to the formation of cytotoxic cell products [1]. The best known feature of HD is neurodegeneration, and this is particularly widespread in the striatal nuclei, basal ganglia and cerebral cortex, resulting in neurological symptoms that involve motor, cognitive and psychiatric disturbances. This leads to a wide-range of clinical features, including personality changes, motor impairment, weight loss and dementia. These symptoms progress over the course of the disease, typically resulting in death after 15–20 years [1]. Currently, HD is a devastating and invariably lethal disease that has no approved treatments to target its molecular cause [2]. Since huntingtin transcripts have been detected in virtually any cell type and tissue [3], HD is being recognised as a multi-system disorder [4,5], affecting a number of major peripheral tissues, including skeletal muscles [6,7,8] and the heart [9,10,11]. Importantly, it has been shown that HTT is also involved in a number of critical cellular processes such as metabolism; protein turnover; regulation of gene expression; and cellular dynamics, such as cytoskeleton endocytosis, trafficking and adhesion (reviewed in [12,13]). This wide range of HTT cellular functions can be explained by interactions with more than 350 interaction partners identified so far [12,14].

Similarly, the ocular tissue has been reported to be affected in HD patients (reviewed in [15]). In fact, the first studies in HD carriers at the pre-symptomatic stage of the disease revealed the following symptoms to be associated with HD: increased blinking, slowness of horizontal saccades, a decrease in saccade speed and an increase in saccade latency (reviewed in [16]). Furthermore, retinal dysfunction has been also detected in HD carriers as they developed higher-than-normal incremental thresholds when tasked to detect blue light as an adaptation to a yellow background [17]. A recently published case study report showed that 25 year-old pre-symptomatic HD patients displayed malfunctions of rod and cone cells in the central retina, based on a visual electrophysiology test [18]. Furthermore, a couple of clinical studies found a reduction in the retinal nerve fibre layer thickness in HD carriers [19,20]. Therefore, HD pathology in the eye warrants further study.

A recent study, both in humans and HD mouse models, discovered significant changes in the cerebrovascular vessel density [21]. In addition, several studies in HD mouse models described HD-related pathological remodelling of the retina, with progressive changes in the photoreceptor layer architecture [22,23]. These studies used two “fragment” HD mouse models, namely R6/2 and R6/1, which are transgenic for the human mutant exon1 HTT fragment. Thus, an analysis of electroretinograms (ERG) showed retinal dysfunction in symptomatic R6/1 mice. This pathological remodelling of the retina was also recapitulated in the R6/2 mouse model as early as at 4 weeks of age, which represents the pre-symptomatic stage of the disease [22]. Another study performed in symptomatic R6/1 mice found a severe deficit in cone response, with an increased level of Muller cells’ gliosis [23]. Such changes were observed in the downstream neuronal cell population in the rod and cone bipolar cells [23]. Retinal dysfunction and its progressive degeneration were also shown in fly models of Huntington’s disease [24].

There is growing evidence of retina-related pathological abnormalities that have already been identified in both pre- and clinical studies. Hence, our current study focuses on structural abnormalities of the optic nerve in HD settings, which have not been explored in detail before. Using very sensitive optical coherence tomography, we aimed to identify any structural changes in the cohort of the pre-symptomatic Polish HD carriers. In addition, we were also interested in discovering any potential changes in the structure of the optic nerve in the well-established R6/2 mouse model at the symptomatic stage of the disease by performing transmission electron microscopy of the murine ocular tissue.

## 2. Material and Methods

### 2.1. Participants

A total of 50 HD carriers from the EHDN (European Huntington’s Disease Network) clinic in Poznan, Poland, were contacted about participating in the study: 13 patients were prospectively enrolled into the study along with 14 age- and sex-matched healthy individuals. All HD carriers were examined clinically for motor, psychiatric and cognitive signs and were scored using the Unified Huntington’s Disease Rating Scale (UHDRS). Only HD carriers with the UHDRS motor scale score < 5 were enrolled into the study, representing the pre-symptomatic stage of the disease, as described previously [25]. Family members of HD patients and others who were negative for HD mutations were enrolled into the healthy control group (14 individuals). All subjects gave their informed consent for inclusion before they participated in the study. The study was conducted in accordance with the Declaration of Helsinki, and the protocol was approved by the Ethics Committee at the Poznan University of Medical Sciences, Poznan, Poland, decision no. 1278/18.

### 2.2. Ophthalmological Pre-Examination and Exclusion Criteria

All participants underwent a thorough ophthalmic examination in order to exclude ocular pathology that could potentially interfere with the OCT analysis. This examination included best-corrected visual acuity measurements and intraocular pressure. Participants with any previous history of ocular disease were excluded from the study. Additionally, the following exclusion criteria were applicable: refractive error > ±3, a high intraocular pressure, a presence of involuntary movements and any medication with known interference on retinal thickness.

### 2.3. Optical Coherence Tomography

The Revo NX 110 (Optopol, Zawiercie, Poland) spectral domain (SD) OCT, equipped with the iTracking technology to compensate for involuntary eye movement and blinks, was used in this study. High-resolution scans measured thickness of the retinal nerve fibre layer (RFNL) and the morphology of the optic disc. The measured area was 3.40 mm within the centre of the optic discs; ring diameter was 0.40 mm and contained 110,000 scans per second. RNFL measurement was performed in four defined sectors. The peripapillary RNFL thickness parameters evaluated in this study were average thickness of a 360° measurement, temporal, nasal, superior and inferior. The GCC retinal ganglion cells were measured within a 7 × 7 mm area divided into six segments. The retina thickness in the macula was measured within a 7 × 7 mm area containing 110,000 scans per second. This area was divided into three paraxial circles measuring 1, 3 and 6 mm in diameter, which were divided into nine defined zones. All scans were obtained by one examiner in a single session.

### 2.4. Mouse Maintenance and Breeding

Hemizygous R6/2 mice were bred by backcrossing R6/2 males to (CBA x C57BL/6) F1 females (B6CBAF1/OlaHsd, Harlan Olac, Bicester, UK), as described previously [26]. All animals had unlimited access to water and breeding chow (Special Diet Services, Witham, UK), and housing conditions and environmental enrichment were as previously described [27]. Mice were subjected to a 12 h light/dark cycle. The animal study protocol was approved by the Home Office, UK, and approved by the Animal Welfare and Ethical Review Body of Imperial College London, project licence number: PAB101FA8.

### 2.5. Genotyping

Genomic DNA was isolated from an ear-punch. R6/2 mice were genotyped by PCR, and the CAG-repeat length was measured as previously described [28]. The cumulative CAG counts for the symptomatic 9-week-old R6/2 mice were 161 ± 3.1 SD. In total, five R6/2 and five control mice were used in this study.

### 2.6. Transmission Electron Microscopy Studies

For electron microscopy (EM) analysis, R6/2 mice and their wild-type littermates were terminally anesthetised by IP injection of 100 µL Lethobarb (sodium pentobarbital). Next, mice underwent transcardial perfusion using a peristaltic pump (at a pressure of 40 mm Hg) with saline. Once the liver was cleared of blood, mice were perfused with a fixative solution containing: 0.1% sodium nitrite, 2% PFA and 2.5% glutaraldehyde in 100 mM PIPES. After the mice were perfused, the whole eye tissue with the optic nerve was dissected from each mouse and kept in the fixative buffer overnight at 4 °C. Next, the eye tissues were transferred into PBS containing 0.1% sodium azide and stored at 4 °C. Samples were then processed for routine transmission electron microscopy. The tissues were washed three times in the 0.1M phosphate buffer, pH 7.2, followed by a fixation with 1% OsO4 in the phosphate buffer, pH 7.2, for 2 h at 4 °C. Next, the globes were washed three times (10 min washes) with the 0.1M phosphate buffer pH 7.2. This was followed by dehydration in a graded ethanol series (40% ± 100% acetone/ethanol and acetone) and finally in aceton. Next, the tissues were infiltrated with propylene oxide-812 resin (Polysciences, Inc., Warrington, PA, USA). The eye tissues were embedded with fresh 100% Epon 812 resins in moulds and polymerised at 60 °C for 48 h. Ultrathin 70 nm sections were prepared using Leica Ultracut UCT (Leica Microsystems, Nussloch, Germany) followed by post embedding with uranyl acetate and lead citrate and analysed using a TEM JEM 1010 (Jeol, Akishima-shi, Japan) electron microscope. All pictograms were taken with a MEGAVIEW G2 Olympus camera.

### 2.7. Optic Nerve and Peripheral Nerve Axon Quantification

The thickness of myelin sheaths was quantified using iTEM Digital Imaging Solution (Olympus Soft Imaging solutions GmbH, Münster, Germany). Briefly, the globes were dissected and a cross section of the optic nerve region (posterior to the globe) was processed for epoxy embedding as described above. Semi-thin (1 μm) sections were used as quality control for representative analysis. Data were collected from over 120 axons for myelin sheath thickness analysis (35–50 pictograms per each studied mouse).

### 2.8. Statistical Analysis

Values are presented as mean ± SD. Statistical analysis was performed using ONE-WAY ANOVA with the Bonferroni post hoc test (InStat software, GraphPad, San Diego, CA, USA). A *p*-value of 0.05 was considered a significant difference. All clinical data were analysed separately for both eyes using the statistical analysis described above.

## 3. Results

Ocular tissue malfunctions often coexist with ongoing central nervous system (CNS) degeneration. Ocular neurodegeneration manifests itself through the pathological remodelling of the retina and alterations in the structure and functionality of the optic nerve. Hence, various CNS pathologies may display visual manifestations, often preceding central symptoms. Since the main pathology of Huntington’s disease is within the CNS, in this study, we aimed to investigate structural changes in the optic nerve in human subjects and in pre-clinical settings by studying the well-established R6/2 mouse model of HD. We employed optical coherence tomography (OCT) in order to study structures of the retina and optic nerve in HD pre-symptomatic gene carriers and healthy individuals. Moreover, we used spectral domain optical coherence tomography (SD-OCT) to also study lesions involving the pre-laminar areas.

This study was conducted on a Polish population of HD carriers and healthy individuals. All participants were of Caucasian ethnicity. The studied population consisted of 13 pre-symptomatic HD patients, based on the Unified Huntington’s Disease Rating Scale (UHDRS). In addition, we examined a cohort of 14 non-HD carriers that were enrolled into a control group. On average, the pre-symptomatic HD patients were 43.1 ± 6.7 SD years old (men and women); the non-HD patients were of similar age to the pre-symptomatic HD patients, at 39.9 ± 8.7 SD years old. There was no statistically significant difference in age between the HD cohort and healthy individuals (*p* = 0.438; one-way ANOVA). The CAG-repeat size in the mutated allele was on average 44.6 ± 4.5 SD while the CAG count in the healthy allele was 17.4 ± 5.1 SD in the pre-symptomatic HD cohort. All patients in the control group were screened and confirmed as non-HD carriers.

**Figure 1:**
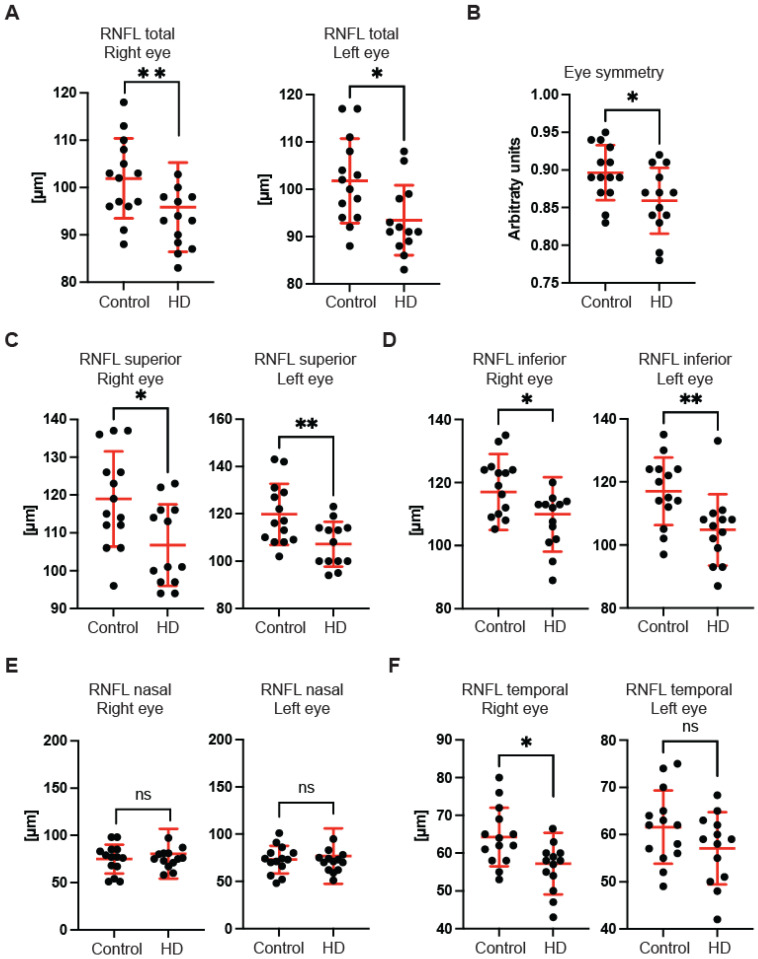
Optical coherence tomography of the retinal nerve fibre layer (RNFL) parameters in HD gene carriers and healthy controls. The following parameters have been assessed in pre-symptomatic HD carriers and age- and sex-matched healthy individuals: (**A**) total RNFL thinning, (**B**) the eye symmetry, (**C**) superior RNFL thinning, (**D**) inferior RNFL thinning, (**E**) nasal RNFL thinning and (**F**) temporal RNFL thinning. Error bars are ± SD (*n* > 13). One-way ANOVA with Bonferroni post hoc test: * *p* < 0.05, ** *p* < 0.01.

**Figure 2:**
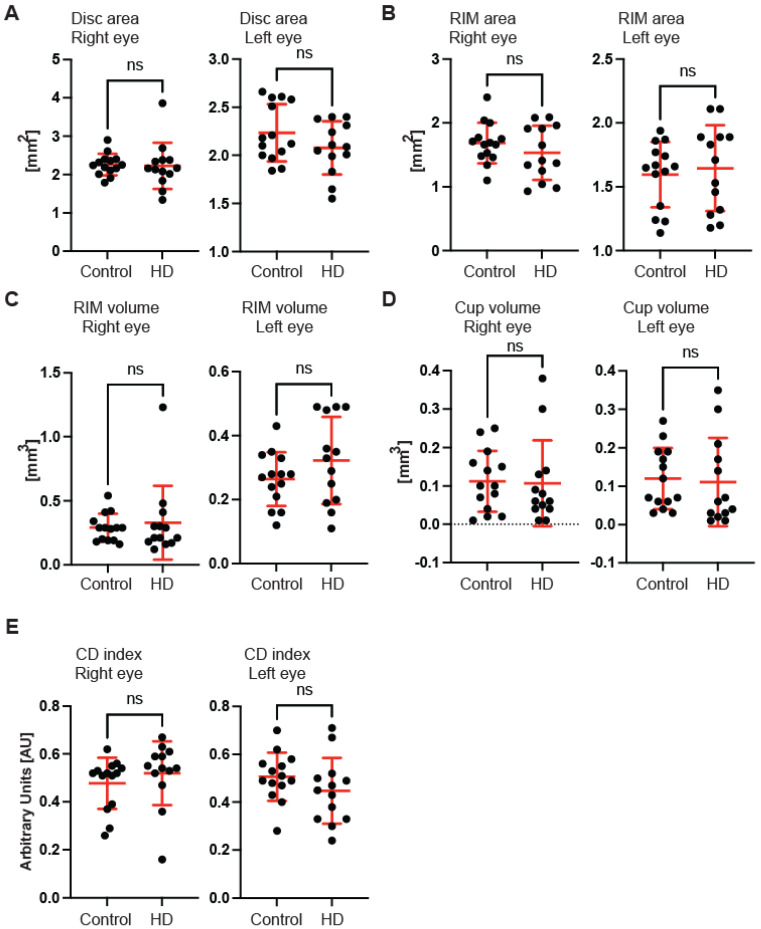
Optical coherence tomography of the retinal nerve fibre layer (RNFL) parameters at the disc level in HD gene carriers and healthy controls. The following parameters have been assessed in pre-symptomatic HD carriers and age- and sex-matched healthy individuals: (**A**) disc area, (**B**) rim area (**C**) rim volume, (**D**) cup volume and (**E**) C/D index. Error bars are ± SD (*n* > 13). One-way ANOVA with Bonferroni post hoc test: C/D, cup-to-disc ratio. ns—non specific.

**Figure 3:**
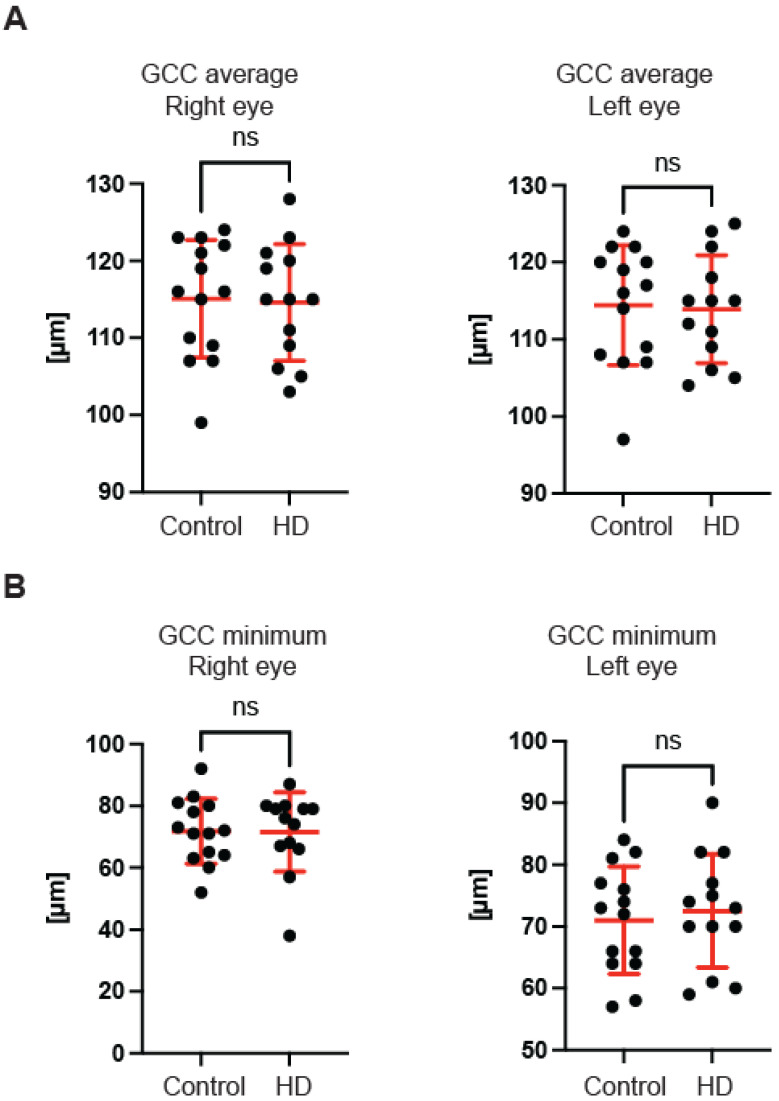
Optical coherence tomography of the ganglion cell complex (GCC) in HD gene carriers and healthy individuals. (**A**) The average (**A**) and minimum (**B**) GCC parameters consisting of three innermost retinal layers: the nerve fibre layer, the ganglion cell layer and the inner plexiform layer. Error bars are ± SD (*n* > 13). One-way ANOVA with Bonferroni post hoc test. ns—non specific.

**Figure 4:**
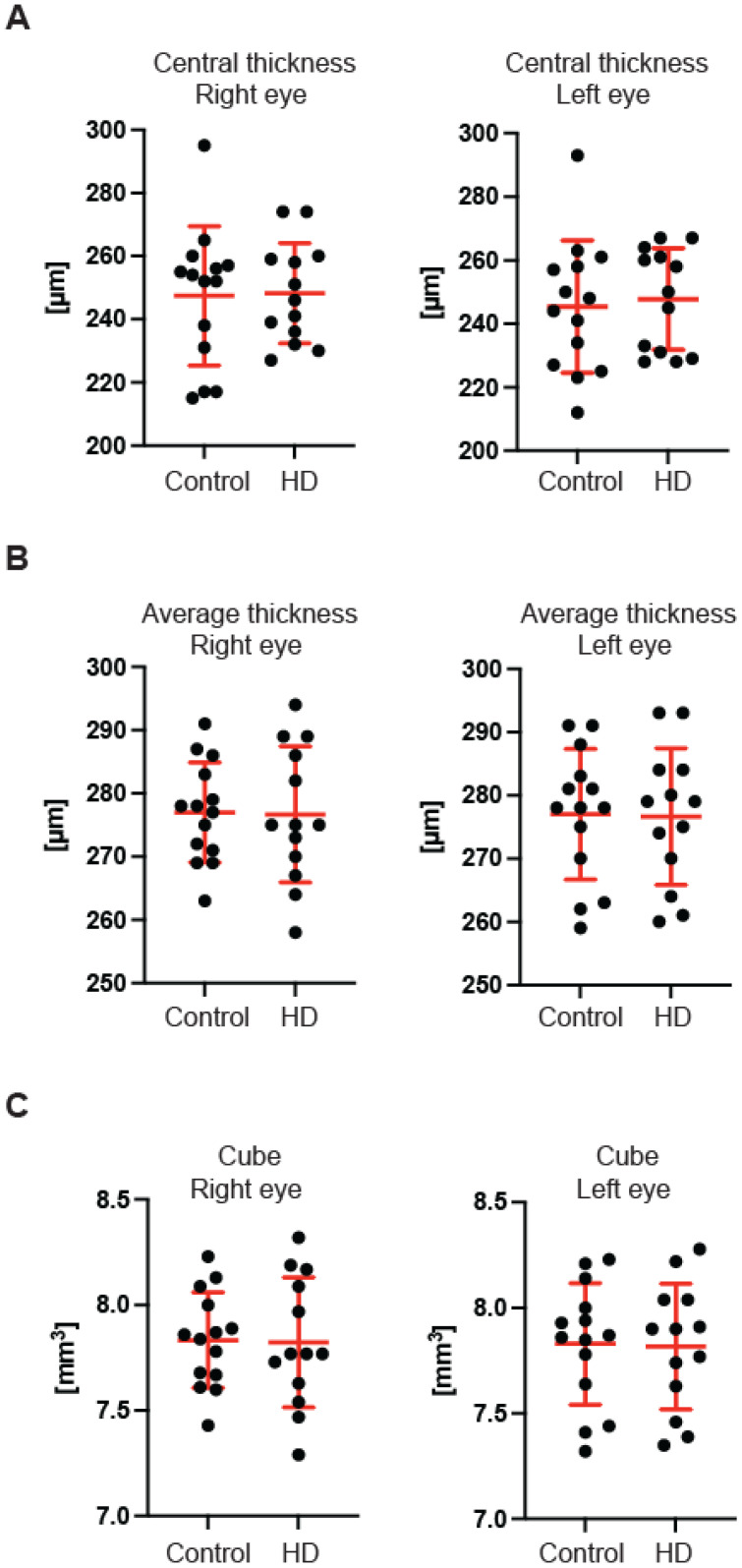
The retina thickness is unchanged in pre-symptomatic HD carriers using OCT. The central (**A**), the average (**B**) and the cube (**C**) volume of the retina from the inner limiting membrane (ILM) to the retinal pigment epithelium (RPE) were unchanged in the HD patients in comparison with the healthy controls. Error bars are ± SD (*n* > 13). One-way ANOVA with Bonferroni post hoc test.

First, we used OCT to capture and quantify axonal loss through measurements of the retinal nerve fibre layer (RNFL) thickness (Figure 1). Retinal nerve fibre layer thickness represents the ganglion cell axons before they enter the optic nerve. We found that the total RNFL thickness was significantly reduced in both left and right eyes of pre-symptomatic HD carriers in comparison with healthy controls (Figure 1A). Consequently, the eye symmetry was significantly reduced in HD patients in comparison with healthy age-matched controls (Figure 1B). The Eye Symmetry Index (ESI) was calculated based on overlapping RNFL measurements in both eyes and represents additional parameters that describe any RNFL structural abnormalities in both eyes. We also detected a significant reduction in RNFL thickness in the specific quadrants such as superior, where a significant reduction in RNFL thickness was found in both eyes (Figure 1C); inferior, where a significant reduction in RNFL thickness was detected in both eyes of HD pre-symptomatic HD patients (Figure 1D); nasal, where no significant changes in RNFL thickness were detected between healthy individuals and HD carriers (Figure 1E); and temporal, where a significant reduction in the RNFL thickness was observed only in the right eye of HD patients in comparison with the control group (Figure 1F). Next, we measured the dimensions of the optic nerve head cup and disc. We found no significant differences between pre-symptomatic HD carriers and the control group, in the disc area (Figure 2A), the rim area (Figure 2B), the rim volume (Figure 2C), the cup volume (Figure 2D) or the cup-to-disc ratio (Figure 2E). Hence, one may conclude that the optic nerve is not affected at the disc level in pre-symptomatic HD patients. We also found no significant differences of the retina thinning at the macula in HD patients in comparison with the control group. The ganglion cell complex (GCC) consists of three innermost retinal layers: the nerve fibre layer, the ganglion cell layer and the inner plexiform layer. There was no significant difference in the average thickness (Figure 3A) and in the minimum thickness (Figure 3B) of the GCC parameter in the pre-symptomatic HD carriers. Finally, we measured the retina thickness from the inner limiting membrane (ILM) to the retinal pigment epithelium (RPE) in the pre-symptomatic HD patients. We found no significant changes of the retina thickness at the central location (Figure 4A) in both eyes of the HD carriers. There were also no significant changes in the average thickness of the retina (Figure 4B) and the cube (Figure 4C) in HD patients. Our comprehensive analysis of the structural changes in the optic nerve in pre-symptomatic HD carriers of the Caucasian descent revealed specific changes in the structure of RNFL in the retina only. Furthermore, the optic nerve at the GCC level and at the disc remained unaffected.

**Figure 5:**
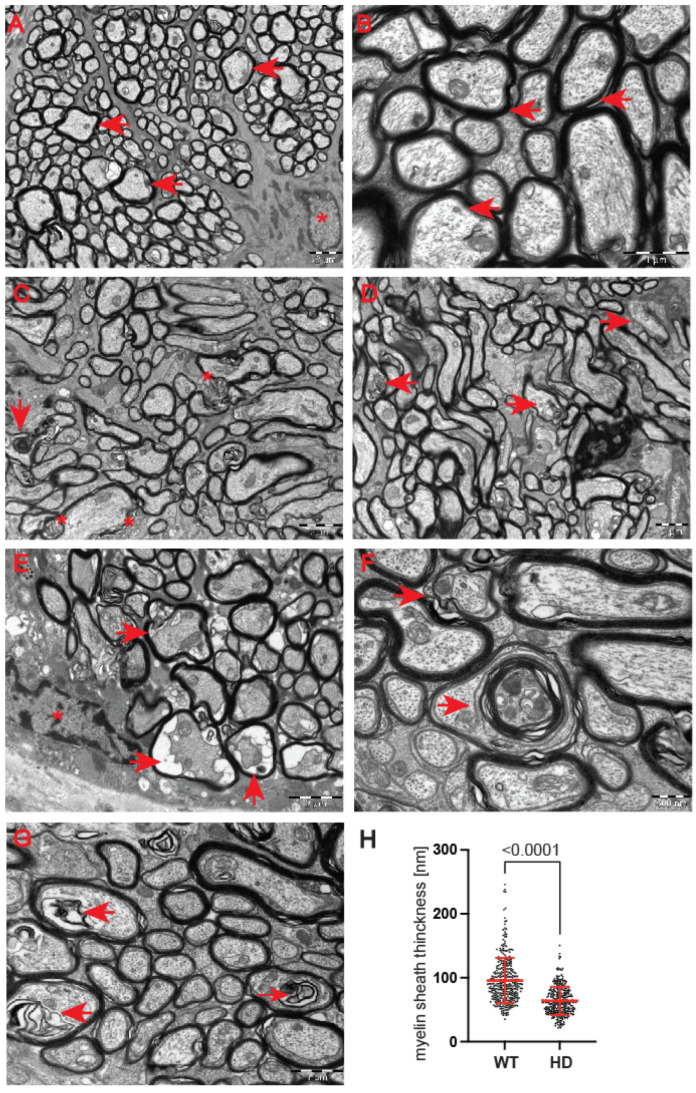
Structural abnormalities of the optic nerve in the symptomatic R6/2 mouse model. Representative electron micrographs of axons in the wild-type mice: (**A**,**B**) control age-matched wild-type mice, the ganglion cell axons displayed a typical morphology with a dense myelin sheath (arrowheads indicate axons; stars indicate astrocytes). The axoplasm contains small regular mitochondria, neurotubules and neurofilaments. (**C**) An abnormal structure of the myelin sheath of axons in the optic nerve in the symptomatic R6/2 mice. An apparent lamellar separation on the myelin layers is shown (asterisk), with an evident presence of myelinoid bodies (arrowhead). (**D**) Examples (arrowheads) of irregularly shaped and demyelinated sheaths of axons in symptomatic HD mice. (**E**) A presence of degenerating axons with collapsed structures and an apparent separation of the myelin sheath are shown (arrowheads) in the R6/2 mice; an oligodendrocyte is marked with an asterisk. (**F**) An example of two sheaths concentrically surrounding the same axon in the HD mice (arrowheads). (**G**) An example of HD axons with plate-like fillings (arrowheads). (**H**) The myelin sheaths of axons are significantly reduced in the symptomatic HD mouse model. Error bars are ± SD (n > 100). One-way ANOVA with Bonferroni post hoc test.

**Figure 6:**
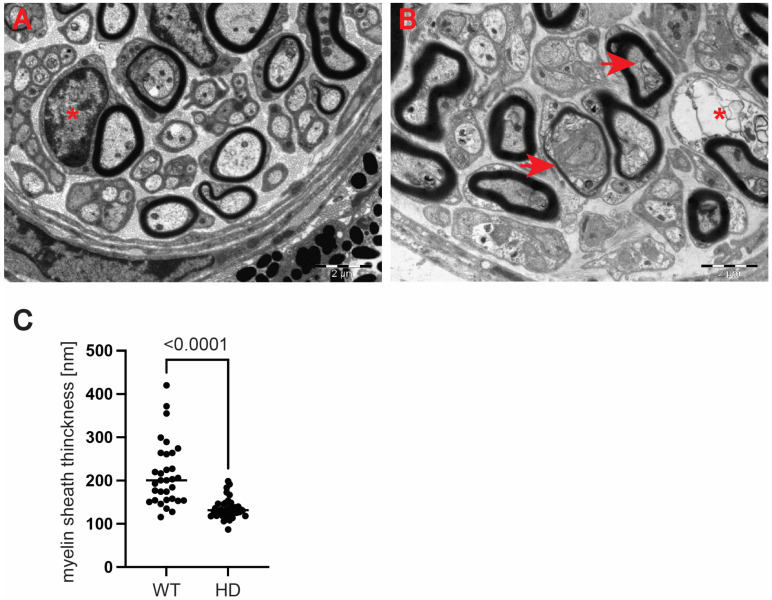
Structural abnormalities of the peripheral nerve structures in symptomatic R6/2 mice. (**A**) A representative electron microscopy pictogram of the choroids area in the wild-type mice. Axons display correct morphology with properly dense myelin sheaths. The cells exhibit a normal distribution and morphology of typical cellular components such as mitochondria, neurotubules and neurofilaments. A typical Schwann cell is indicated with an asterisk. (**B**) A representative electron pictogram from the choroids area in the HD mouse model. An abnormal Schwann cell is presented with a degenerated cytoplasm with an apparent oedema and vacuolisation (asterisk). HD axons display progressive degeneration of the myelin sheath with apparent shrinkage of axons (arrowheads). (**C**) A significant reduction in the myelin sheaths’ thickness in the symptomatic R6/2 mice in comparison with their littermates. Error bars are ± SD (n > 100). One-way ANOVA with Bonferroni post hoc test.

Next, we used a transmission electron microscopy approach to identify pathological remodelling of the optic nerve in the well-established R6/2 mouse model of Huntington’s disease. The R6/2 mouse model, which is transgenic for a mutated N-terminal Exon 1 HTT fragment, recapitulates a number of structural, molecular, physiological and behavioural pathological abnormalities. These overlap with characteristics of adult onset human HD but occur in the mouse within approximately 12–16 weeks of age [29]. In our study, we used the whole ocular tissue isolated from 9-week old R6/2 mice and age-matched wild-type controls. This corresponds to a symptomatic stage of Huntington’s disease with an apparent presence of mutant Huntingtin aggregates and significant changes in a number of neurological and behavioural tests, as described previously [28]. At the ultrastructural level, we found structurally affected myelin sheaths of axons in the symptomatic R6/2 mice (Figure 5C–G) in comparison with wild-type littermates (Figure 5A,B). One of the main characteristics of affected HD axons was lamellar separation of the myelin layers, with appearance of myelonoid bodies (Figure 5C). Furthermore, these axons displayed demyelinated sheaths (Figure 5D), often with collapsed cellular structures (Figure 5E). Another structural abnormality of HD axons was the co-centric appearance of two sheaths (Figure 5F), accompanied with plate-like fillings (Figure 5G). Finally, we found that the thickness of the myelin sheaths of axons in the symptomatic R6/2 mice were significantly reduced in comparison with the control group (Figure 5H). We also investigated whether structural alterations of axons are only restricted to the optic nerve or whether peripheral nerves are similarly affected. We discovered that Schwann cells showed a number of indications of degenerating cells, such as oedema and vacuolisation in the HD mouse model (Figure 6B) in comparison with healthy littermates (Figure 6A). Similar to the optic nerve, axons at the choroids area showed thickening of the myelin sheaths (Figure 6B). Our quantitative analysis showed that indeed there was a significant reduction in myelin thickness in the symptomatic R6/2 mice when compared with the wild-type mice (Figure 6C). Hence, our ultrastructural analysis revealed for the first time that the optic nerve structure is significantly altered in symptomatic HD mice. This is in line with our discovery of the affected retinal nerve layers in the pre-symptomatic human HD carriers based on our optical coherence tomography data.

**Figure 7:**
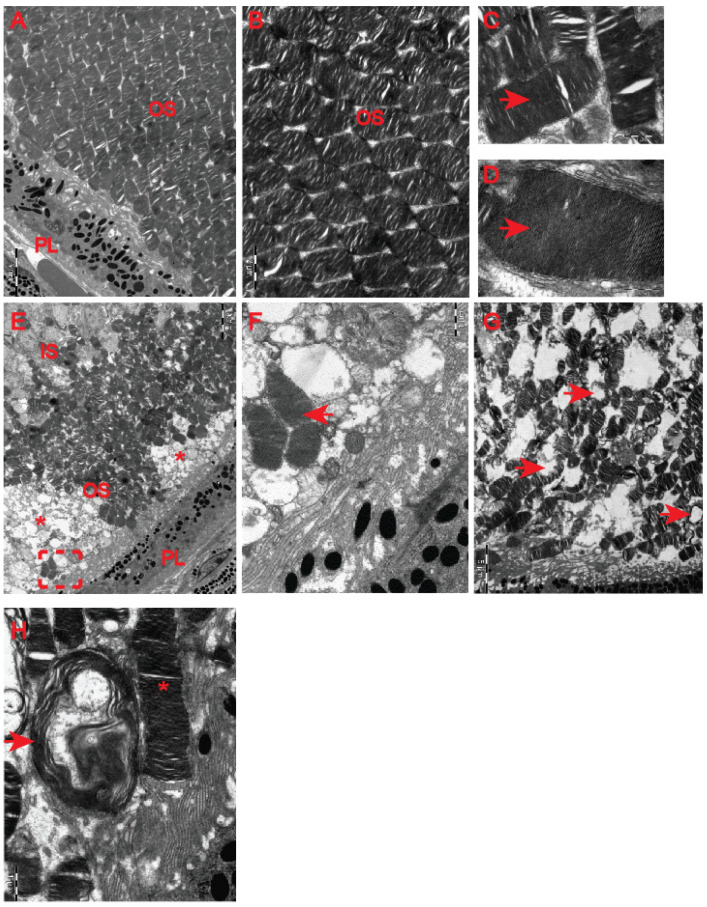
HD-related structural abnormalities in the retina. Representative electron micrographs of the retina in control age-matched mice (**A**–**D**) and in the symptomatic HD mice (**E**–**G**). (**A**,**B**) A typical structure of the light-sensitive outer segment (OS) containing the rod and cone photoreceptor cells, PL—pigment epithelium. (**C**,**D**) A higher magnification of electron microscopy pictograms representing regularly distributed columns containing parallel membrane disks (arrowheads) with visual pigments. (**E**) An example of the outer segment (OS), containing photosensitive cells, displays a number of areas undergoing disintegration (asterisk) (IS—inner segment, PL—pigment epithelium). (**F**) A higher magnification of the area from (**E**) displaying correctly structured segments of the photosensitive cells (overheads) in the symptomatic HD mice. (**G**) An apparent vacuolisation and swelling of photoreceptor cells containing damaged membrane disks (arrowheads). (**H**) A higher magnification of damaged membrane (arrowheads) and the correct discs (asterisk).

**Figure 8:**
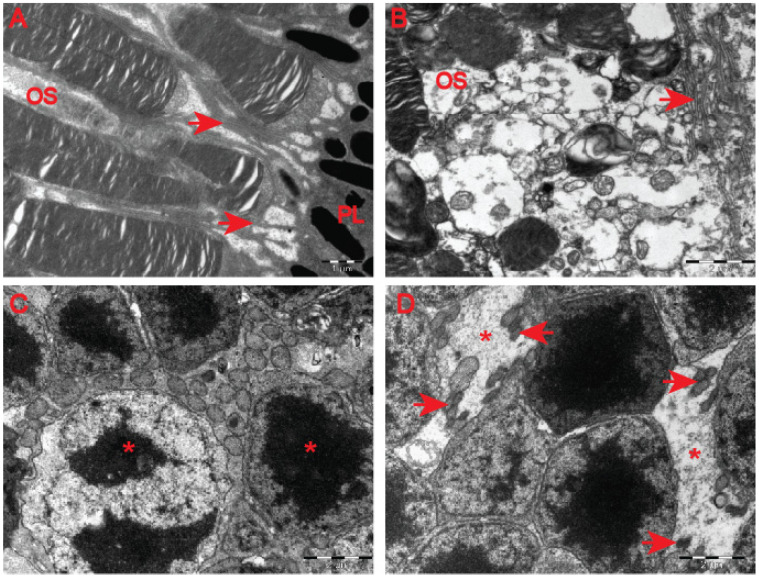
Structural changes in the outer retina of the symptomatic HD mouse model. (**A**) A typical morphology of the microvilli (arrowheads) that arises from the apical membrane of the pigment epithelial cells surrounding outer segments (OS) of photoreceptor cells in wild-type mice. (**B**) An example of degenerative changes within the microvilli (arrowheads) of the apical pigment of the epithelium in the symptomatic HD mouse model. (**C**) A regular structure of photoreceptor nuclei (asterisks) located in the ONL layer of the wild-type mice. (**D**) An example of the ONL (outer nuclear layer) layer structure that displays extracellular empty spaces between nuclei of photoreceptor cells (asterisks). Atrophied axonal projections labelled with arrowheads. ONL—outer nuclear layer.

Although our clinical OCT data did not indicate that pre-symptomatic HD carriers developed retinal abnormalities (Figure 4), earlier studies in HD mouse models [22,23] revealed HD-related structural remodelling of the retina based on electroretinogram analysis and histological examination. Hence, we used electron microscopy to unravel ultrastructural changes in the retina of the symptomatic R6/2 mice. We found that photoreceptor cells contained damaged membrane disks with apparent vacuolisation and swelling (Figure 7F–H) in comparison with the healthy age-matched control group (Figure 7A–D). Often, HD photoreceptors within the outer segment of retina layers showed a progressive disintegration, likely leading to cell death (Figure 7E). In fact, we also observed that a number of photoreceptor cells in the outer retina displayed degenerative characteristics within their microvilli in the symptomatic R6/2 mice (Figure 8B) in comparison with the wild-type controls (Figure 8A). The outer retina also underwent pathological structural remodelling in the symptomatic R6/2 mice in comparison with their wild-type littermates. The outer nuclear layer (ONL) of the retina showed atrophied axonal projections with a number of intracellular empty spaces (Figure 8D) in comparison with a regular structure of ONL in healthy wild-type mice (Figure 8C). Hence, our ultrastructural study of the HD retina identified a number of structural events highlighting the pathological remodelling of HD retina.

## 4. Discussion

Recently, ocular dysfunction has been linked to many neurodegenerative diseases including Alzheimer’s disease (AD), Parkinson’s disease (PD) and Huntington’s disease (HD) [15]. Such a link could be explained by the fact that neurons in both the central nervous system and in the ocular tissue arise from the developing neural tube. The mammalian retina is composed of various types of cells including photoreceptors, amacrine cells and ganglion cells responsible for the final transmission of visual signals to the brain [30]. Hence, it has become apparent that ocular tissue could also be affected in those diseases. Huntington’s disease is an inherited genetic, fatal disorder classified as a rare disease with a prevalence in the general population of 1 in 10,000 [1]. HD is caused by a clearly defined mutation (which is the opposite to the major two neurodegenerative diseases of AD and PD). The HD mutation occurs within the exon-1 of huntingtin HTT gene [29], which is ubiquitously expressed in all cell types and tissues [3,31], including ocular tissue [22]. HD mouse models have begun to be used to study HD-related retinopathy and significant structural changes have been identified in the retina [22,23].

In this study, we investigated the structure of the optic nerve in clinical settings in pre-symptomatic HD carriers by using coherence optical tomography. Furthermore, in pre-clinical settings, we used the R6/2 mouse model of HD and applied an ultra-sensitive approach based on transmission electron microscopy. We found that pre-symptomatic HD carriers showed a unique pattern of structural changes in the retinal nerve fibre layer. We identified that the total RNFL thickness was significantly reduced. The thinning of RNFL in specific quadrants (superior, inferior and temporal) was also significantly affected. Interestingly, we did not find any abnormalities in the GCC region and at the macular level in HD pre-symptomatic patients. Our findings are in agreement with two previously published studies in an Argentinian HD cohort [19] and in Australian HD patients [20] as both studies reported RNFL thinning. However, the study by Gatto et al. showed significant differences in the temporal and superior RNFL [19], while another report by Kersten et al. noticed significant abnormalities only in the temporal RNFL [20]. Hence, one might conclude that the RNFL thinning identified so far in three different ethnic groups might be considered as potentially useful biomarkers. For example, one could use these observations to monitor the effectiveness of HD gene therapies. Moreover, for the first time, we analysed OCT scans for each globe separately, and this allowed us to identify significant changes in the eye symmetry of HD carriers. Interestingly, the RNFL thinning has also been described in other neurodegenerative diseases, including Alzheimer’s disease. AD patients showed a significant thickness reduction in global and temporal superior quadrants in the peripapillary RNFL [32]. Moreover, PD patients also displayed RNFL thinning [33], and a significant reduction in the RNFL thickness has been observed in the inferior sector [34]. This quadrant was also affected in our study in HD pre-symptomatic carriers. Furthermore, Friedreich’s ataxia (FRDA) patients displayed statistically significant thinning of average retinal nerve fibre layer (RNFL) and thinning in all but the temporal quadrant [35]. Finally, a comprehensive study was performed using optical coherence tomography in spinocerebellar ataxia carriers: SCA-1, SCA-2, SCA-3 and SCA-6 [36]. It showed that (RNFL) thickness was reduced for patients with SCA-2 and SCA-3, and thickness in the macular region was reduced for all SCAs except for SCA2. SCA-2 carriers showed only RNFL thinning, which mirrors our current study in HD carriers and might indicate that both polyglutamine diseases (SCA-2 and HD) display similar clinical abnormalities in SD-OCT scans. The main limitation of this study is the relatively small number of participants that could be enrolled. However, our stringent exclusion criteria significantly limited the number of HD participants. In addition, it should be emphasised that symptomatic HD carriers (especially those with advanced involuntary movements) were excluded from the study due to the nature of the SD-OCT technology. Finally, we excluded any patients undergoing or having completed any gene therapy clinical trial.

We were also interested in investigating any ultra-structural changes in the optic nerve in pre-clinical settings using the R6/2 mouse model. This model has historically been widely used in a number of pre-clinical validations of potential therapeutic targets. Our study revealed for the first time, at the ultrastructural level, that HD photoreceptor cells contained severely damaged membrane disks with evident vacuolisation and swelling. Moreover, the outer segment of retina layers showed a progressive disintegration that is in line with previously published studies [22,23]. Importantly, the transmission electron microscopy approach revealed alterations in the myelin sheaths of axons with a lamellar separation of the myelin and presence of myelonoid bodies. There was a significant thinning of myelin sheaths in the optic nerve and in peripheral nerves. Schwann cells showed a number of features characteristic to degenerating cells such as oedema and vacuolisation. Such changes might indicate a potential mitochondrial dysfunction, which has been already widely described in other HD tissues such as skeletal muscles [6,26] and in HD-related cardiomyopathy [9,10,11,37,38]. Interestingly, some of the changes observed by us and others in the temporal RFNL in pre-manifestation HD carriers have also been seen in other mitochondriopathies such as Friedreich’s ataxia [39].

It would be important to investigate the ultrastructural changes in other HD mouse models next, including so-called “full length” models such as *Hdh*Q150, in which the CAG expansion has been introduced into the endogenous mouse Htt locus. Hence, a novel set of biomarkers that would allow for monitoring HD-related pathological remodelling of the retina and the optic nerve in different HD mouse models would be of great importance, especially in the context of validation of novel gene therapy technologies. This strategy might be also applicable to a wider spectrum of other polyglutamine diseases, which are very poorly studied in pre-clinical studies. In fact, we could only find one study in a spinocereberal ataxia 7 (SCA-7) knock-in model that described a significant shortening of photoreceptor outer segments [40].

Ultimately, identifying biomarkers of HD pathology in the eye both will lead to insights into the aetiology of HD and will help to benchmark new therapies.
